# BIOPSYCHOSOCIAL DETERMINANTS OF PHYSICAL ACTIVITY IN BLACK WOMEN WITH CARDIOVASCULAR DISEASE

**DOI:** 10.1093/geroni/igae098.3474

**Published:** 2024-12-31

**Authors:** Hyun Jung Kim, Shweta Gore

**Affiliations:** MGH Institute of Health Professions, Boston, Massachusetts, United States; MGH Institute of Health Professions, Boston, Massachusetts, United States

## Abstract

Cardiovascular disease (CMD) remains the primary cause of death among Black women in the United States. Given the well-recognized benefits of physical activity (PA) in reducing CVD mortality, PA levels among Black women remain notably low. This study aims to explore factors associated with low PA in Black women with CMD via secondary analysis of subjective Global Physical Activity Questionnaire data in the 2013-2028 National Health and Nutrition Examination Survey (NHANES) sample. The prevalence of CVD among Black women (26,100,000 ± 2,171,143) in the estimated total population of United States (US) was significantly greater than in Black men (21,300,000 ± 1,592,102) (t = -5.02, p < 0.001). Significantly greater proportion of Black women in the US did not meet the recommended PA guidelines (79.9% versus 89.3% in Black men and women; t = 3.51, p = 0.001). Among Black men and women, age, self-perceived health, and difficulty walking emerged as significant predictors of meeting PA guidelines (F5, 40 = 10.07, p < 0.001, whereas age and high glycosylated hemoglobin (HbA1c) were significant predictors among Black women (F8, 36 = 2.62, p = 0.023). Every unit increase in age and HbA1c decreased the odds of Black women meeting the PA guidelines by 5% and 27.6% (OR = 0.96, p = 0.014 and OR = 0.96, p = 0.014).

